# Management of Dermoid Cysts During Pregnancy: A Retrospective Analysis From a Tertiary Care Center

**DOI:** 10.7759/cureus.104356

**Published:** 2026-02-27

**Authors:** Aarti G Suryawanshi, Rajni Agrawal, Archana Bharti, Kanupriya Verma, Sharmila, Deepshikha Verma

**Affiliations:** 1 Obstetrics and Gynaecology, Maharaja Agrasen Medical College, Hisar, IND; 2 Anesthesiology, Maharaja Agrasen Medical College, Hisar, IND

**Keywords:** dermoid cyst, laparoscopy, maternal-fetal outcomes, ovarian torsion, pregnancy complications

## Abstract

Background

Dermoid cysts, or mature cystic teratomas, are the most common benign ovarian neoplasms in women of reproductive age and are frequently encountered during pregnancy. Their management poses unique challenges, requiring a balance between maternal-fetal safety and risks of surgery. Evidence from Indian tertiary care centers remains limited. This study aimed to evaluate the clinical profile, management strategies, and maternal-fetal outcomes of dermoid cysts in pregnancy.

Methods

This retrospective observational study included 87 pregnant women diagnosed with dermoid cysts over six years at Maharaja Agrasen Medical College Agroha, Hisar (Haryana), a tertiary care center in North India. Demographic data, clinical presentation, ultrasonographic findings, cyst characteristics, management approach, operative details, and maternal and perinatal outcomes were analyzed. Comparisons between conservatively and surgically managed groups were performed using Chi-square/Fisher’s exact test and Student’s t-test as appropriate.

Results

The mean maternal age was 27.8 ± 4.6 years, and the majority of patients were diagnosed during the second trimester 55 (63.2%), predominantly through routine antenatal ultrasonography. The mean cyst size was 6.8 ± 2.7 cm, with torsion observed in 9 (10.3%) of cases. Conservative management was employed in 53 (60.9%), while 21 (24.1%) required antenatal surgery, predominantly laparoscopic cystectomy in the second trimester. Intraoperative complications were rare, and no malignant transformations were observed on histopathology. The mean gestational age at delivery was 38.1 weeks, with preterm birth in 14 (16.1%) of cases. Perinatal outcomes, including birth weight, NICU admission, and mortality, were comparable between conservatively and surgically managed groups.

Conclusion

Most dermoid cysts in pregnancy can be safely managed expectantly. When surgery is indicated, laparoscopic intervention during the second trimester is safe and associated with favorable maternal and fetal outcomes. These findings support a risk-stratified, individualized management approach, particularly relevant in resource-constrained settings.

## Introduction

Dermoid cysts, or mature cystic teratomas, are the most common benign germ cell tumors of the ovary, accounting for approximately 10-20% of all ovarian neoplasms [1]. Composed of well-differentiated derivatives of the three germ layers, these lesions are typically slow-growing and are most frequently encountered in women of reproductive age [1]. Their prevalence during pregnancy makes them clinically significant, as they represent the most common ovarian mass identified in this population. The reported incidence of adnexal masses in pregnancy ranges from 0.2% to 2%, with dermoid cysts constituting nearly one-third of these cases [2].

Management during pregnancy poses a unique clinical challenge because most dermoid cysts are asymptomatic and incidentally detected during routine antenatal ultrasonography [3]. However, potential complications, such as ovarian torsion, rupture, infection, or, rarely, malignant transformation, may occur during pregnancy. Ovarian torsion is the most frequent complication, reported in approximately 3-10% of cases, particularly in the first and early second trimesters, whereas rupture and infection are uncommon, and malignant transformation is exceedingly rare (<2%) in pregnancy. The risk of torsion is highest in the first and early second trimesters, making early detection and timely intervention essential [4].

Historically, conservative management was preferred to avoid surgical and anesthetic risks, particularly in early pregnancy. Advances in anesthesia safety and minimally invasive surgical techniques have shifted practice toward individualized, risk-based management [5]. Laparoscopic cystectomy, particularly when performed during the second trimester, is now widely considered a safe and effective approach for managing ovarian dermoid cysts in pregnancy. Several studies have demonstrated that laparoscopy is associated with lower maternal morbidity, reduced postoperative pain, shorter hospital stay, and faster recovery when compared with laparotomy, without increasing the risk of miscarriage, preterm labor, or adverse perinatal outcomes. Large case series and systematic reviews report low rates of intraoperative complications (1-6%), minimal conversion to laparotomy (<5-8%), and no significant increase in fetal loss or preterm delivery following laparoscopic surgery during pregnancy. A 14-year single-center experience further supports the safety and feasibility of laparoscopic cystectomy in pregnancy, demonstrating favorable maternal and fetal outcomes with careful patient selection and appropriate surgical expertise [6].

Recent evidence supports this evolving approach. It is reported that 95% of dermoid cysts were incidentally diagnosed, with 70% managed conservatively without adverse outcomes, while surgically managed cases were mostly treated laparoscopically with favorable results [7]. Similarly, it is found that low torsion rates (3.2%) and no significant difference in perinatal outcomes between affected and unaffected pregnancies [8]. It is also demonstrated that laparoscopic elective surgery reduces hospital stay and lowers the risk of preterm birth compared to emergency intervention [9].

Given persistent uncertainty regarding optimal timing and surgical approach, particularly in resource-limited settings, data from tertiary care centers are invaluable [10]. This study aimed to evaluate the clinical profile, management strategies, and maternal-fetal outcomes of dermoid cysts in pregnancy to inform evidence-based practice.

## Materials and methods

Study design and setting

This retrospective observational study was conducted in the Department of Obstetrics and Gynecology at Maharaja Agrasen Medical College Agroha, Hisar (Haryana), a tertiary care teaching hospital in North India, which serves as a referral center for a large obstetric population. The study included pregnant women diagnosed with dermoid cysts over a six-year period from January 2018 to December 2023. Approval was obtained from the Institutional Ethics Committee prior to data collection (IEC Approval No.: MAMC/IEC/2024/043). As the study was retrospective and record-based with no direct patient contact, the requirement for informed consent was waived.

Study population

The study population comprised all pregnant women diagnosed with ovarian dermoid cysts during the antenatal period who subsequently delivered at our institution within the study duration. Diagnosis was primarily based on ultrasonographic features characteristic of mature cystic teratoma, including echogenic mural nodules (Rokitansky nodule), hyperechoic lines and dots suggestive of hair, and fat-fluid levels. In patients who underwent surgical management, the diagnosis was further confirmed intraoperatively and by histopathological examination. Patients with adnexal masses of other etiologies, such as functional ovarian cysts, endometriomas, or suspected malignant ovarian tumors, were excluded. Cases with incomplete medical records or missing outcome data were also excluded from the analysis.

Investigations and tumor marker assessment

Routine laboratory investigations were performed as part of standard antenatal care. Tumor marker evaluation, specifically serum CA-125, was not performed routinely in all patients. CA-125 estimation was carried out selectively in cases with larger cyst size (≥8 cm), atypical or complex ultrasonographic features, rapid increase in cyst size, or acute presentation where malignancy could not be confidently excluded. Routine assessment of tumor-specific germ cell markers (such as alpha-fetoprotein, beta-human chorionic gonadotropin, or lactate dehydrogenase) was not undertaken, as dermoid cysts were diagnosed with high confidence based on classical imaging features, and there was no clinical or radiological suspicion of malignant germ cell tumors. In addition, the diagnostic utility of these markers during pregnancy is limited due to physiological alterations.

Data collection

Data were retrieved from hospital electronic medical records, antenatal case files, operative registers, and histopathology reports. Collected variables included maternal age, parity, antenatal registration status, gestational age at diagnosis, presenting symptoms, mode of detection, ultrasonographic characteristics, laterality, and maximum cyst diameter. Details regarding management strategy, i.e., expectant or surgical, timing and indication for surgery, surgical approach (laparoscopy or laparotomy), intraoperative findings, perioperative complications, and histopathological results were documented. In patients managed conservatively, records of serial ultrasonographic follow-up and changes in cyst size or morphology were reviewed.

Outcome measures

Primary maternal outcomes included complications related to dermoid cysts such as ovarian torsion, rupture, infection, need for emergency surgical intervention, and perioperative morbidity. Perinatal outcomes assessed were gestational age at delivery, mode of delivery, neonatal birth weight, Apgar scores at one and five minutes, requirement for neonatal intensive care unit (NICU) admission, and perinatal morbidity or mortality. Cases where cystectomy was performed concurrently with cesarean section were analyzed separately to assess intraoperative feasibility and outcomes.

Statistical analysis

Data were entered into Microsoft Excel (Microsoft Corp., USA) and analyzed using IBM SPSS Statistics for Windows, version 20.0 (released 2011, IBM Corp., Armonk, NY). Continuous variables were expressed as mean ± standard deviation (SD) or median with interquartile range (IQR), as appropriate. Categorical variables were summarized as frequencies and percentages. Comparative analysis between conservatively and surgically managed groups was performed using Student’s t-test or Mann-Whitney U test for continuous variables and Chi-square or Fisher’s exact test for categorical variables. A p-value <0.05 was considered statistically significant.

Ethical considerations

The Institutional Ethics Review Board (IERB) of Maharaja Agrasen Medical College, Agroha, reviewed the research proposal, study documents, and data collection format during its meeting held in February 2024 and granted approval for the study.

## Results

The mean maternal age of the study cohort (n = 87) was 27.8 ± 4.6 years, with the majority (36; 41.4%) in the 26-30-year age group. More than half of the patients were multigravidae (46; 52.9%), and ANC registration status showed that 69 (79.3%) women were registered for antenatal care, reflecting regular follow-up and access to routine obstetric ultrasonography, which is crucial for early detection, surveillance, and timely management of adnexal masses during pregnancy. The mean gestational age at diagnosis was 18.9 ± 6.8 weeks, with the majority diagnosed during the second trimester (55; 63.2%). Most cases were detected incidentally on routine ultrasound (72; 82.8%), with only 15 (17.2%) presenting symptomatically. Anemia was the most frequent comorbidity (21; 24.1%), followed by hypothyroidism (8; 9.2%) and gestational diabetes mellitus (6; 6.9%) (Table 1).

**Table 1:** Baseline demographic and obstetric characteristics of patients with dermoid cysts in pregnancy (n = 87). Hb: hemoglobin; GDM: gestational diabetes mellitus; PIH: pregnancy-induced Hypertension; ANC: antenatal checkup

Variable	Frequency (%)/mean ± SD
Maternal age (years)	27.8 ± 4.6
Age groups (years)	
<20	6 (6.9)
21–25	24 (27.6)
26–30	36 (41.4)
31–35	17 (19.5)
>35	4 (4.6)
Parity	
Primigravida	41 (47.1)
Multigravida	46 (52.9)
ANC registration status	
Registered	69 (79.3)
Not registered	18 (20.7)
Gestational age at diagnosis (weeks)	18.9 ± 6.8
Trimester at diagnosis	
First trimester	18 (20.7)
Second trimester	55 (63.2)
Third trimester	14 (16.1)
Mode of detection	
Routine ultrasound	72 (82.8)
Symptomatic presentation	15 (17.2)
Symptoms at presentation	
Acute pain (torsion)	7 (8.0)
Dull ache	6 (6.9)
Abdominal mass/distension	2 (2.3)
Comorbidities	
Anemia (Hb <11 g/dL)	21 (24.1)
Hypothyroidism	8 (9.2)
GDM	6 (6.9)
PIH	5 (5.7)

Right-sided dermoid cysts were more common (49; 56.3%) than left-sided (34; 39.1%), with bilateral involvement in four (4.6%). The mean cyst diameter was 6.8 ± 2.7 cm, with nearly half measuring between 5 and 8 cm. Classical sonographic features such as Rokitansky nodules (61; 70.1%) and echogenic lines/dots (54; 62.1%) were frequently observed. Complications at presentation were uncommon, with torsion suspected in nine (10.3%) and rupture in one (1.1%). Tumor markers were assessed selectively in 31 (35.6%) patients based on clinical and radiological suspicion. CA-125 estimation was performed in cases with larger cyst size (>8 cm), atypical or complex ultrasonographic features, rapid increase in size, or acute symptoms suggestive of complications. Routine tumor marker evaluation was not performed in asymptomatic patients with classical sonographic features of dermoid cysts, in accordance with standard practice, given the limited diagnostic utility of CA-125 in pregnancy and its physiological elevation during gestation (Table 2).

**Table 2:** Ultrasonographic and intraoperative characteristics of dermoid cysts in pregnancy (n = 87). USG: ultrasonography; CA-125: cancer antigen 125; *non-mutually exclusive; $CA-125 was assessed selectively in a subset of patients where adnexal masses demonstrated large size, atypical ultrasonographic features, or acute symptoms, primarily to aid in exclusion of epithelial ovarian malignancy. Tumor-specific germ cell markers were not routinely evaluated, as dermoid cysts are typically diagnosed with high confidence based on characteristic ultrasonographic features, and routine germ cell marker testing is not recommended in asymptomatic cases with classical imaging findings during pregnancy.

Variable	Frequency (%)/mean ± SD
Laterality	
Right	49 (56.3)
Left	34 (39.1)
Bilateral	4 (4.6)
Maximum diameter (cm)	6.8 ± 2.7
Size categories	
<5 cm	22 (25.3)
5–8 cm	41 (47.1)
>8 cm	24 (27.6)
Number of cysts	
Single	83 (95.4)
Multiple	4 (4.6)
Classic dermoid USG features	
Rokitansky nodule	61 (70.1)
Echogenic lines/dots	54 (62.1)
Fat–fluid level	28 (32.2)
Complications at presentation	
Suspected torsion	9 (10.3)
Rupture	1 (1.1)
Infection	2 (2.3)
Tumor marker checked (CA-125)$	31 (35.6)
Elevated >35 U/mL	5 (5.7)
Associated pelvic pathology	
Fibroid uterus	6 (6.9)
Contralateral simple cyst	7 (8.0)

More than half of the cases (53; 60.9%) were managed conservatively (expectant 49 (56.3%), including elective postpartum surgery 4 (4.6%)), with close antenatal follow-up. Antenatal surgery was required in 21 patients (24.1%), predominantly via laparoscopy (16; 76.2%). Most surgeries were performed during the second trimester (18; 85.7% of antenatal procedures). Thirteen patients (13; 14.9%) underwent cystectomy during cesarean section. Torsion (9; 42.9%) was the most common indication for surgery, followed by persistent pain and rapid cyst growth (Table 3).

**Table 3:** Management strategies for dermoid cysts in pregnancy (n = 87). LSCS: lower-segment cesarean section; IQR: interquartile range; *percentages for laparoscopy and laparotomy calculated relative to those undergoing surgery during pregnancy (n = 21).

Variable	Frequency (%)/median (IQR)
Management approach	
Conservative (expectant including elective postpartum surgery)	49 (56.3)
Surgery during pregnancy	21 (24.1)
• Laparoscopy	16 (76.2)*
• Laparotomy	5 (23.8)*
Surgery at cesarean section	13 (14.9)
Indication for antenatal surgery (n = 21)	
Torsion	9 (42.9)
Persistent/worsening pain	6 (28.6)
Rapid growth / suspicious features	5 (23.8)
Rupture	1 (4.7)
Trimester of antenatal surgery (n = 21)	
First trimester	2 (9.5)
Second trimester	18 (85.7)
Third trimester	1 (4.8)
Median postoperative stay (days)	
Laparoscopy	3 (2–4)
Laparotomy	5 (4–7)

Of the 34 surgically managed patients, 10 (29.4%) underwent emergency procedures. Conversion from laparoscopy to laparotomy was required in one case (6.3% of laparoscopic surgeries). Intraoperative complications were uncommon, with two patients (5.9%) experiencing significant hemorrhage exceeding 500 mL, both of whom required transfusion. Postoperative complications were observed in six patients (17.6%), with fever (3; 8.8%) and wound infection (2; 5.9%) being the most frequent, followed by postoperative ileus in one case (2.9%). Prolonged hospitalization (>5 days) was noted in seven (20.6%) surgically managed patients. Histopathological examination confirmed mature cystic teratoma in all 34 cases (100%), and no malignant transformation was identified (Table 4).

**Table 4:** Maternal intra- and postoperative outcomes among surgically managed patients (n = 34).

Variable	Frequency (%)
Emergency surgery	10 (29.4)
Conversion from laparoscopy to laparotomy (n = 16 laparoscopies)	1 (6.3)
Intraoperative complications	
Hemorrhage > 500 mL	2 (5.9)
Postoperative complications	
Fever	3 (8.8)
Wound infection	2 (5.9)
Ileus	1 (2.9)
Blood transfusion required	3 (8.8)
Prolonged hospital stay (>5 days)	7 (20.6)
Histopathology result	
Mature cystic teratoma	34 (100)
Malignant transformation	0 (0.0)

The mean gestational age at delivery was 38.1 ± 1.9 weeks, with preterm delivery observed in 14 (16.1%) cases. Vaginal delivery was the most common mode (51; 58.6%), while 36 (41.4%) required cesarean section. The mean birth weight was 2.79 ± 0.46 kg, with low birth weight noted in 21 (24.1%) neonates. Perinatal outcomes were generally favorable, with low rates of Apgar <7 at five minutes (4; 4.6%), NICU admission (11; 12.6%), and perinatal mortality (1; 1.1%) (Table 5).

**Table 5:** Perinatal outcomes of pregnancies complicated by dermoid cysts (n = 87). LSCS: lower-segment cesarean section; NICU: neonatal intensive care unit

Variable	Frequency (%)/mean ± SD
Gestational age at delivery (weeks)	38.1 ± 1.9
Preterm delivery (<37 weeks)	14 (16.1)
Mode of delivery	
Vaginal delivery	51 (58.6)
Elective LSCS	14 (16.1)
Emergency LSCS	22 (25.3)
Birth weight (kg)	2.79 ± 0.46
Low birth weight (<2.5 kg)	21 (24.1)
Apgar <7 at five minutes	4 (4.6)
NICU admission	11 (12.6)
Perinatal morbidity	8 (9.2)
Perinatal mortality	1 (1.1)
Congenital anomalies (unrelated)	1 (1.1)

Patients undergoing surgical management had significantly larger cysts compared to those managed expectantly (7.9 ± 2.9 cm vs. 6.1 ± 2.2 cm; p = 0.001) and were more likely to present with acute symptoms, although this did not reach statistical significance (p = 0.062). Rates of preterm delivery were slightly higher in the surgical group (8 (23.5%) vs. 6 (11.3%)), although not statistically significant (p = 0.121). Perinatal outcomes, including birth weight and NICU admission, were comparable between the two groups, suggesting that timely surgical intervention does not adversely affect fetal outcomes (Table 6).

**Table 6:** Comparison of clinical and perinatal outcomes between conservatively and surgically managed patients (n = 87). SD: standard deviation; NICU: neonatal intensive care unit

Variable	Conservative (n = 53)	Surgical (n = 34)	p-value
	Frequency (%)/mean ± SD		
Cyst size (cm)	6.1 ± 2.2	7.9 ± 2.9	0.001
Symptomatic at presentation	6 (11.3)	9 (26.5)	0.062
Torsion	0 (0)	9 (26.5)	<0.001
GA at delivery (weeks)	38.3 ± 1.7	37.8 ± 2.1	0.124
Preterm birth <37 weeks	6 (11.3)	8 (23.5)	0.121
NICU admission	5 (9.4)	6 (17.6)	0.242
LSCS	15 (28.3)	21 (61.8)	0.002
Postop complications (maternal)	—	5 (14.7)	—

## Discussion

This retrospective study of 87 pregnant patients with dermoid cysts contributes valuable real-world data to the growing literature on adnexal masses in pregnancy. The mean maternal age of 27.8 years and predominance of cases in the 26-30-year age group are consistent with prior studies by Saleh et al. and Yayla Abide et al., indicating that mature cystic teratomas occur most commonly in women of reproductive age [11,12]. The majority of patients in our study were multigravidae (52.9%) and booked cases (79.3%), reflecting a population with reasonable antenatal surveillance, which likely facilitated early detection. The finding that nearly two-thirds were diagnosed in the second trimester mirrors the reports by Martone et al. and Osto et al., where mid-trimester anomaly scans accounted for the majority of detections [13,14].

The mean cyst size in our series was 6.8 ± 2.7 cm, which falls within the range reported in studies by Almaazmi et al. and Njoku et al. [15,16]. Notably, cysts >8 cm were associated with higher rates of surgical intervention, supporting the observation by Walid et al. that larger adnexal masses pose a greater risk of torsion and persistent pain [17]. Our torsion rate (10.3%) was slightly higher than that reported in studies by Tankou et al. and Hodges et al. (2-8%), likely reflecting the tertiary care referral bias and the inclusion of symptomatic patients [8,18]. Importantly, rupture and infection were rare, consistent with prior findings by Saleh et al. that most dermoid cysts remain uncomplicated during pregnancy [11].

Management decisions in this study were individualized based on cyst size, symptomatology, and gestational age. Conservative management was employed in 56.3% of cases, with no adverse outcomes reported, corroborating the evidence that asymptomatic dermoid cysts <6 cm may be safely observed with serial ultrasonography [13,16]. Surgical intervention was required in approximately one-quarter of patients, predominantly in the second trimester, which is widely considered the safest window for non-obstetric surgery due to reduced risk of miscarriage and preterm labor [19].

A notable strength of our series is the high utilization of laparoscopy (76.2% of antenatal surgeries). Laparoscopic management is increasingly favored, and several studies have demonstrated its safety and feasibility in pregnancy, with lower intraoperative blood loss, shorter hospital stay, and faster recovery compared to laparotomy Cavaco-Gomes et al., and Moradan et al., [20,21]. Our conversion rate to laparotomy (6.3%) and overall intra- and postoperative complication rate (8.8%) are comparable to, or lower than, those reported in published series, where conversion rates range from 5-10% and overall complication rates range from 5-15% for laparoscopic management of adnexal masses during pregnancy, thereby supporting the safety and feasibility of laparoscopy in appropriately selected patients. Moreover, there were no cases of malignant transformation, echoing the study by Li et al., that malignancy rates in dermoid cysts during pregnancy are extremely low (<2%) [22].

Perinatal outcomes in our cohort were reassuring. The mean gestational age at delivery was 38 weeks, with preterm delivery observed in 16.1%-a rate slightly higher than baseline population rates but comparable to literature by Hakoun et al., evaluating adnexal masses in pregnancy [23]. Birth weight, NICU admission rates (12.6%), and perinatal mortality (1.1%) were all within acceptable ranges and comparable to those reported by Tankou et al. [18]. Importantly, we found no statistically significant difference in perinatal outcomes between conservatively and surgically managed groups, suggesting that timely surgical intervention, when indicated, does not adversely affect fetal well-being. These findings are consistent with the study by Surampudi et al., indicating that elective surgery is not associated with increased miscarriage, preterm delivery, or growth restriction [24].

From a pathophysiological standpoint, our findings underscore the importance of balancing maternal-fetal risks [25]. The higher proportion of torsion in early gestation supports the theory that rapid uterine growth leads to displacement and twisting of adnexal structures [26]. The preference for laparoscopic surgery in the second trimester is biologically and technically sound, as the gravid uterus is still small enough to allow good visualization while minimizing the risk of first-trimester teratogenicity or third-trimester preterm contractions [27].

Strengths and limitations

The present study has several notable strengths. It represents a relatively large single-center experience from India, a region where data on the management of adnexal masses in pregnancy remain limited. Comprehensive documentation of demographic, clinical, ultrasonographic, operative, and perinatal variables allowed for an integrated assessment of both maternal and fetal outcomes. Importantly, the inclusion of patients managed conservatively as well as surgically enabled meaningful comparison between management strategies. The high proportion of laparoscopic interventions adds contemporary relevance, particularly in the context of evolving evidence supporting minimally invasive surgery during pregnancy in resource-limited settings.

Nonetheless, certain limitations should be acknowledged. The retrospective design is inherently subject to information bias and dependence on the accuracy and completeness of medical records. As a tertiary referral center, our institution may receive a higher proportion of complicated or symptomatic cases, introducing potential selection bias. Long-term neonatal and developmental outcomes were not evaluated, limiting assessment of the possible subtle effects of intraoperative intervention or anesthesia exposure during pregnancy. In addition, tumor marker evaluation, including CA-125, was performed selectively based on clinical and radiological suspicion rather than routinely, precluding comprehensive analysis of their diagnostic or prognostic utility in this cohort.

Future directions

Future research should aim at prospective, multicenter studies or registries to enhance generalizability, reduce selection bias, and standardize diagnostic and management protocols. Comparative studies employing matched or randomized designs to evaluate laparoscopic versus open surgical approaches would provide higher-level evidence regarding maternal and perinatal safety. Further work is also required to establish evidence-based thresholds for surgical intervention based on cyst size, growth dynamics, and imaging characteristics, particularly in low- and middle-income settings where access to advanced laparoscopic expertise may be variable. In addition, cost-effectiveness analyses may help inform institutional and policy-level decisions regarding optimal management strategies in resource-constrained healthcare systems.

## Conclusions

Dermoid cysts represent the most common ovarian masses encountered during pregnancy, and the majority can be managed conservatively with careful antenatal surveillance. Our findings indicate that when surgical intervention is warranted, laparoscopic cystectomy, preferably performed during the second trimester, is feasible and associated with low maternal morbidity and acceptable perinatal outcomes. Management decisions should be individualized, taking into account cyst size, symptomatology, and gestational age. This risk-stratified approach is particularly relevant in resource-limited tertiary care settings and provides institution-specific evidence to support informed clinical decision-making and guide future practice recommendations.
